# Low-dose radiation therapy for idiopathic or interstitial cystitis in male cats

**DOI:** 10.1093/jvimsj/aalaf029

**Published:** 2026-01-21

**Authors:** Allison Kendall, Shelly Vaden, Tracy Gieger, Tonya Harris, Margaret E Gruen, Michael W Nolan

**Affiliations:** Department of Clinical Sciences, North Carolina State University, Raleigh, NC 27606, United States; Department of Clinical Sciences, North Carolina State University, Raleigh, NC 27606, United States; Department of Clinical Sciences, North Carolina State University, Raleigh, NC 27606, United States; Department of Clinical Sciences, North Carolina State University, Raleigh, NC 27606, United States; Department of Clinical Sciences, North Carolina State University, Raleigh, NC 27606, United States; Department of Clinical Sciences, North Carolina State University, Raleigh, NC 27606, United States; Comparative Medicine Institute, North Carolina State University, Raleigh, NC 27606, United States

**Keywords:** cystitis, feline, radiotherapy; bladder

## Abstract

**Background:**

Idiopathic cystitis (IC) accounts for the majority of lower urinary tract (LUT) disease in cats and is characterized by recurrent clinical signs or urethral obstruction (UO), presenting ongoing challenges in clinical management.

**Hypothesis/Objectives:**

Determine the feasibility of using single-fraction low-dose radiotherapy (RT) to reduce rate of re-obstruction and recurrence of clinical signs in cats with feline idiopathic cystitis.

**Animals:**

Fifteen client-owned male cats with recent history of severe IC and historical UO that remained symptomatic despite environmental modification and pharmacological management.

**Methods:**

An IACUC-approved, single-arm, single-institution, prospective clinical trial was conducted to evaluate the clinical effects of irradiating the entire LUT with a single 6 Gy fraction.

**Results:**

One cat was immediately lost to follow-up, and 1 died 516 days after RT. The remaining 13 cats were alive at the time of data analysis, with a median follow-up of 548 (range 70-1307) days. All but 1 had symptomatic improvement. Six had a single flare-up of signs of IC at a median of 243 days after RT (range 4-395 days). After RT, 1 cat had a recurrent UO, which occurred at 11 months and was managed surgically.

**Conclusions and clinical importance:**

In this cohort of severely affected cats, >90% had apparent improvement in clinical signs after RT, with no documented adverse effects, demonstrating that in addition to environmental modification, RT is a promising tool for managing IC in male cats.

## Introduction

Idiopathic cystitis (IC) affects between 55% and 69% of cats with lower urinary tract disease (LUTD).^1–3^ Clinical signs of IC include pollakiuria, stranguria, hematuria, and urethral obstruction (UO). The pathogenesis is poorly understood but thought to involve chronic activation of the central threat response system (CTRS), which leads to disruption of the glycosaminoglycan layer of the urinary tract, inflammation, and dysregulation of the neuroendocrine and immune systems, all leading to pain and urethral muscle spasm.^3^^,^^4^ Feline idiopathic cystitis (FIC) likely represents a complex and multifactorial disease process, stemming from both genetic and environmental influences. Even with treatment, clinical signs of FIC recur in 58%-65% of cases.^1^^,^^4^ The most severe clinical sign is UO, and 5%-23% of cats with UO are humanely euthanized at time of obstruction.^5–8^ If cats survive their first episode of UO, 73.5% will have persistent signs of LUTD^9^ and up to 36% will re-obstruct, often within days of initial hospitalization.^5^

Strategies that help suppress activity of the CTRS have been associated with a reduction in clinical signs of FIC, whereas treatments that overlook this aspect tend to be less successful. In the absence of more targeted therapies to restore normal CTRS function, minimizing input to this system through effective multimodal environmental modification (MEMO) has been shown to significantly alleviate clinical signs of FIC and its associated comorbidities.^10^^,^^11^ Despite the implementation of appropriate MEMO, only a few novel therapies for chronic pain and inflammation have been investigated, none of which have been particularly successful.^12–16^ Frequent administration of oral medications in cats also cause anxiopathy, activation of the CTRS, and disruption of the human–animal bond. Chronic use of traditional systemic anti-inflammatory medications in cats can result in adverse effects, including the development or worsening of renal disease with no documented benefit in disease management.^13^

Due to the limited effectiveness of current pharmacological treatments for IC, alternative management approaches are needed. A candidate that has not previously been explored for FIC is low-dose radiotherapy (RT), which provides potent anti-inflammatory and analgesic benefits without systemic adverse effects for a variety of non-neoplastic diseases (eg, osteoarthritis, meningoencephalitis of unknown origin).^17–19^ The purpose of this descriptive study was to evaluate low-dose RT as a strategy for reducing the rate of urethral re-obstruction and the recurrence of clinical signs in cats diagnosed with FIC.

## Materials and methods

The study was approved by the Institutional Animal Care and Use Committee, and written informed consent was obtained from the owner of each animal.

### Animals

Cats were recruited for the study from the surrounding geographical area or presented through the authors’ hospital. To ensure inclusion of a group with clinical disease, study enrollment was limited to male cats with a history of multiple recurrent episodes of FIC-associated clinical signs or UO. This selection was intended to capture a subset of cats with a more severe and persistent disease course, representing the individuals most difficult to manage in clinical practice. Cats with UO or clinical signs attributable to other causes, including urolithiasis, neoplasia, or toxicosis, were excluded. Idiopathic cystitis flares were defined as signs of LUTD lasting for more than 1 day and requiring visits to their primary care veterinarian or requiring visits to their primary care veterinarian. Cats were specifically enrolled, if appropriate MEMO, pharmacological, and dietary therapy had been attempted, and clients were considering perineal urethrostomy (PU) or humane euthanasia. Exclusion criteria included female sex, identified urethral tear, spontaneous (non-iatrogenic) rupture of the urinary bladder, lower urinary tract (LUT) neoplasia, urolithiasis identified by diagnostic imaging, underlying neurologic disease identified as the etiopathogenesis for UO, trauma, or existing PU. Additionally, due to anesthetic safety concerns, cats were excluded if hyperkalemia (>5.5 mmol/L), tachycardia (>220 bpm), hypotension (<90 mmHg systolic), or azotemia (serum creatinine > 5 mg/dL) could not be resolved during hospitalization for initial medical stabilization. All medical records were reviewed, and final enrollment decision was done by the principal investigator (PI) (A.K.).

A detailed client history, body weight, and signalment were recorded for each cat. All cats underwent physical examinations and preanesthetic blood testing consisting of a complete blood count (CBC), serum biochemistry, urinalysis (UA), and aerobic bacterial urine culture. Additionally, either an abdominal radiograph, including urethra or urinary tract ultrasonography was performed at the discretion of the PI to rule out other causes of LUT disease (such as urolithiasis or neoplasia).

Clients were counseled to maintain their cat’s existing home environment, diet, and medications without modification throughout the study period, allowing each cat to serve as its own intrinsic control.

### Radiation therapy

All cats were anesthetized. Induction and maintenance protocols were determined on a case-by-case basis by the attending anesthesiologist, but cats were generally induced with intravenous anesthesia and maintained on gas anesthesia.

**Figure 1:**
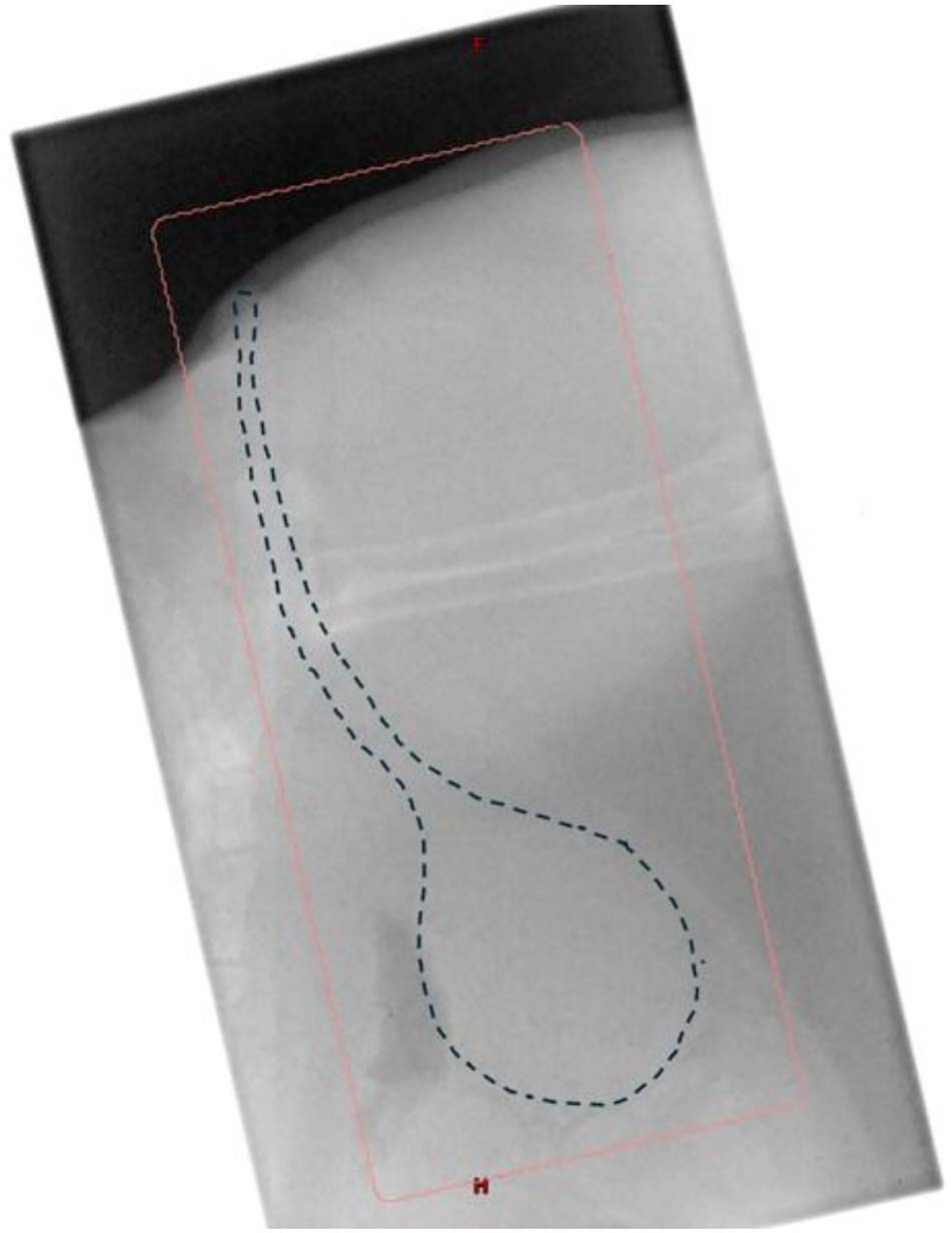
Radiotherapy was delivered using 6 MV photons from a clinical linear accelerator with a manual setup. Cats were positioned in lateral recumbency, and 2 equally weighted, parallel-opposed fields were collimated to encompass the lower urinary tract with a 1 cm margin. Positioning was verified using a lateral megavoltage port film. The dotted line indicates the urinary bladder and urethra; the pink box outlines the radiation field. A bolus was applied to the perineum (after portal imaging) to ensure dose buildup at the urethral orifice and achieve lateral electronic equilibrium. A single 6 Gy fraction was prescribed, and delivered at 600 MU/min.

Radiation therapy (RT) was planned manually, with a lateral abdominal radiograph to aid in mapping out the field (Figure 1). Cats were positioned in lateral recumbency, with the entire LUT in the field and as much normal tissue excluded as possible, using manual blocks. Neither wedges nor bolus were used. Equally weighted parallel-opposed 6 MV X-ray beams were used to deliver a total of 6 Gy in a single fraction. This dose was prescribed to the treatment isocenter, which was located at the midplane along the central axis. Second check software (RadCalc; Lifeline Software, Inc.) was used to verify accuracy of the dose calculation. Megavoltage port films were used to verify correct positioning, and radiation was delivered at 600 MU/min using a standard clinical linear accelerator (Novalis TX, Varian Medical Systems).

### Follow-up

A published client questionnaire^1^ on LUT signs was administered to the owners at baseline, then every 30 days thereafter for up to 1 year. Follow-up beyond 1 year continued via telephone or email updates.

After completion of the study, a behavioral questionnaire designed by the NC State Behavior Service was administered to the owners retroactively via Qualtrics. An amendment was approved by the Institutional Animal Care and Use Committee.

All cats remained under the same management and environmental conditions throughout the study period, enabling each cat to serve as its own internal control for comparison of outcomes before and after treatment.

### Statistical analysis

Sample size was determined using a Simon Minimax Phase II single arm clinical trial design, with a type I error rate of 0.1, power of 80%, 76% response probability for standard therapy, and 90% response probability for the proposed therapy.^20^

Due to the small sample size and exploratory nature of this study, only descriptive statistics were utilized. Categorical data were summarized using counts and percentages, while continuous variables were reported as medians and ranges. No inferential statistical tests were performed. Descriptive outcomes were used to assess clinical signs, owner-reported behavior changes, and treatment responses after low-dose RT. All analyses were conducted using standard spreadsheet software.

## Results

A total of 15 male cats were included in the study. All cats were castrated and represented a variety of coat coloration patterns including tuxedo (5), brown tabby (4), black (3), orange tabby (2), and gray (1). The median age at time of presentation was 4 years (10 months to 10 years), and the median weight was 5.15 kg (3.83-7.24 kg).

All 15 included cats were documented as having recurrent episodes of FIC and at least 1 UO. Cats diagnosed with FIC clinical signs in the 12 months before study enrollment (8 cats) had an average of 2.4 flares monthly. Cats diagnosed with FIC clinical signs for greater than 1-year before enrollment (7 cats) had an average of 3.5 flares yearly.

Before enrollment, 1 cat had 4 episodes of UO, whereas 2 cats had 3 episodes each, 4 cats had 2 episodes each, and 8 cats had a single episode of UO (0 days to 6 years before presentation) (Table S1). One cat received RT during an active episode of UO; the RT was delivered after urinary catheter placement and successful unblocking. Two cats received RT within 1 week after hospitalization for a UO. The remaining 12 cats were recruited from primary care veterinarians, with a maximum interval of 6 months between last UO and RT.

All cats’ owners were determined to have followed appropriate MEMO based on record review by the PI and a previously published LUT questionnaire.^1^ All 15 cats were on a prescription urinary diet at the time of enrollment. Eleven of the 14 cats were receiving daily pain management, NSAID therapy, and behavioral medications, or behavioral medications. Cats were treated with gabapentin (10), prazosin (7), robenacoxib (6), antimicrobial therapy (5), buprenorphine (4), fluoxetine (4), amitriptyline (4), or some combination of those at time of enrollment.

All cats had normal preanesthetic CBC and serum biochemistry. The most common UA abnormality was hematuria with 5-10 (2), 10-15 (2), 15-20 (1), 20-30 (2), >100 (2), and >500 RBC/hpf (2). Seven cats had pyuria [rare WBC/hpf (3), 0-5 WBC/hpf (3), and >100 WBC/hpf (1)]. At the time of evaluation, 1 cat exhibited pyuria alone, 5 cats had hematuria, 6 cats demonstrated both pyuria and hematuria, and 3 cats had neither finding. Only 2 cats had triple phosphate crystals described as few and moderate. None of the 15 cats had bacterial growth in urine specimens submitted for aerobic culture via sterile cystocentesis.

All cats had either abdominal radiographs including urethra or urinary tract ultrasonography reviewed by an ACVR board-certified veterinary radiologist. None of the cats had uroliths or another documented cause of LUT signs.

After treatment with RT, 1 cat was lost to follow-up at 70 days and 1 cat died of an unrelated cause at 516 days after-RT. The remaining 13 cats were alive at the time of manuscript preparation, with a median follow-up time of 548 days (17-1307 days).

### Time to urethral re-obstruction

One cat had a recurrent UO during the RT follow-up period (Table S1). This episode occurred 11 months after treatment, after a known stressor (owner absence), and bacteriuria was documented on UA. The cat had been free of LUT signs until this event, which required hospitalization and surgical management with PU. After RT, this cat had 2 documented UO episodes and an average of 1 FIC flare per month.

### Frequency of LUT signs

Overall, 13 of the 14 cat (93%) owners with follow-up more than 1 year reported an improvement in their LUT signs and were satisfied with the effects of treatment. Six cats (43%) experienced a single flare-up of FIC signs at a median of 243 days after RT (range 4-395 days) (Table S1). One cat had a flare-up at 5 months, was retreated (6 Gy × 1) and remained asymptomatic 540 days later (ie, at the time of manuscript preparation). The other 5 cats with flare-ups after RT were managed medically and had no subsequent clinical signs.

Seven of the 14 cats (50%) have had no reported recurrence of LUT signs during the entire length of follow-up (Table S1).

### Adverse events

Three cats developed leukotrichia over their hind limb at the site of irradiation. At the time of manuscript preparation, no other adverse events had been documented. One cat was euthanized 516 days after RT due to a presumptive colonic tumor. This cat was euthanized prior to advanced diagnostics, and no necropsy was performed; therefore, the presumptive diagnosis could not be confirmed, and its relationship to the radiation treatment field cannot be confirmed or excluded.

### Pharmacological and dietary management

Of the 14 cats with available follow-up data, fluoxetine administration was continued in 3 cats (21%) based on owner preference, while the remaining 11 cats (79%) were not receiving any pharmacologic therapy or supplements at the end of the follow-up period. Six cats (43%) continued to be fed a prescription urinary diet, also due to owner preference, whereas 8 cats (57%) were transitioned to a standard feline adult maintenance diet.

### Behavior assessment

Fourteen of 15 owners completed the behavioral questionnaire. In the assessment of comfort and pain, 11 owners reported that their cats appeared more active and comfortable, 2 observed no change, and 1 reported decreased activity and comfort, though the owner attributed the change to aging rather than treatment. Regarding tolerance to handling, 4 owners noted improvement in their cat’s response to being picked up, while 10 observed no change. Eight owners reported increased positive response to petting, with 6 noting no change. When evaluating tail carriage, 6 owners observed their cats holding their tails higher compared to before treatment, while 8 reported no difference. Over-grooming behavior decreased in 7 cats, remained unchanged in 4, worsened in 1, and 2 owners did not provide a response.

In the evaluation of energy and enthusiasm after treatment, 10 owners reported that their cats appeared more energetic and enthusiastic, 3 noted no change, and 1 observed decreased energy and enthusiasm. Playfulness increased in 10 cats, remained unchanged in 3, and decreased in 1 cat, though the owner attributed the change to aging rather than treatment. Additionally, 11 owners reported increased use of perches and resting areas after treatment, while 3 observed no change.

When assessing overall happiness and contentment after treatment, 12 owners reported that their cats appeared happier and more content, while 2 owners noted no change. Increased affection toward humans in the household was reported by 6 owners, and 5 owners observed increased affection toward other pets in the home.

## Discussion

This study demonstrates that low-dose RT is a safe and effective management option for male cats with IC. After RT, cats had less frequent signs of LUTD and demonstrated improved signs of behavior, as documented by clinical observations and owner-completed questionnaires. The low number of adverse effects observed in this cohort supports the safety of this modality, while the reduction in recurrence of clinical signs and UOs suggests therapeutic benefit. These findings highlight the potential of low-dose RT as an adjunctive treatment strategy for FIC, especially in cases refractory to conventional medical and environmental management.

Radiation therapy is a common treatment for various cancers; however, lower doses of RT are routinely used to effectively alleviate severe pain, as is the case in canine osteosarcoma^21^^,^^22^ and low doses of radiation are potently and rapidly anti-inflammatory. This has allowed veterinarians to use low-dose RT for effective management of various inflammatory and pain conditions, including lymphoplasmacytic rhinitis, lick granulomas,^23^^,^^24^ meningoencephalitis of unknown origin,^25^ and osteoarthritis.^17^^,^^18^ Cats with IC have evidence of chronic inflammatory cell infiltrates and higher numbers of mast cells in the submucosa than healthy cats.^26^ The exact mechanisms that underlie radiation’s anti-inflammatory and analgesic effects are largely unknown, and likely multifactorial. In vitro studies identify several mechanisms underlying the anti-inflammatory effects of RT, notably the modulation of cytokine production and adhesion molecule expression in activated endothelial cells and leukocytes. Additionally, radiation increases nitric oxide production and reduces oxidative bursts in activated macrophages and native granulocytes.^27^^,^^28^ There is greater nitric oxide production in cats with IC,^28^ but data on specific cytokines, adhesion molecules, and their roles in pathogenesis remain limited and are not yet well defined.

In human medicine, the use of low-dose RT for managing chronic inflammatory conditions has declined, due to concerns over unknown long-term, dose-dependent adverse effects, and the availability of more targeted non-steroidal anti-inflammatory drugs (NSAIDs). However, in parts of Europe, particularly Germany, RT for non-neoplastic disease remains more widely practiced and accounts for a notable proportion of the clinical caseload.^29^ In feline medicine, the long-term daily use of NSAIDs is associated with well-documented adverse effects, particularly involving renal and gastrointestinal systems. Moreover, the necessity of daily oral administration can increase stress in cats, which might further exacerbate the underlying disease process in FIC. After RT, 11 of the cats did not require continuation of long-term pain medications or NSAIDs.

The overall improvements in behavior, tail carriage, and affection support the theorized chronic pain and inflammation associated with cystitis. Studies indicate that women with interstitial cystitis/bladder pain syndrome (IC/BPS) experience intense and widespread pain, with pain being a central aspect of their experience.^30^ Additionally, the chronic nature of IC/BPS and its associated pain can lead to psychological distress, with higher incidences of depression and anxiety among affected women.^31^ This is likely true in cats too as most owners reported an improvement in overall happiness and increased affection toward people and pets in the home.

Limitations of this study include a small sample size of a single sex, lack of control group and standardized environmental management, and unknown long term adverse effects beyond a median of 548 days. Due to the novel treatment approach, this study was designed to establish proof-of-concept for low-dose RT and to evaluate its safety protocol and efficacy in a small cohort. Based on the single-arm design, the study was powered to detect a 14% improvement in response rate over standard therapy (from 76% to 90%).^32^ The observed response rate of 93% exceeded this predefined threshold, indicating that low-dose RT might confer a clinically meaningful benefit compared to standard medical management.

To minimize variability and strengthen internal validity, each cat served as its own intrinsic control. Owners were instructed to maintain all conventional and environmental management strategies used before low-dose RT, and compliance was monitored through structured questionnaires. This approach aimed to isolate the effects of RT by reducing confounding variables related to changes in medical or environmental management. The late addition of the behavioral questionnaire was a result of spontaneous owner-reported improvements in behavioral signs. The retrospective nature of the structured questionnaire could have introduced recall bias and future prospective studies will evaluate behavioral questionnaires in real time to minimize this potential.

Ongoing follow-up of the cats enrolled in this study will continue beyond the preparation of this manuscript to monitor for potential long-term adverse effects of low-dose RT. While only leukotrichia was noted as a late-effect complication, the development of radiation-induced neoplasia remains a theoretical concern. Although the total dose delivered was low (6 Gy), the use of a single large fraction confers different biologic effects compared to more protracted courses of 0.5-2 Gy per fraction. At this dose level, the risk of late toxicity, such as stricture, perforation, or radiation-induced neoplasia, is considered low but not negligible. Most risk estimates for radiation-induced cancer are extrapolated from data involving high or moderate doses of RT, using the linear no-threshold model, which assumes that even the smallest dose carries some risk. However, radiation-induced neoplasia is considered a stochastic event and its risk at low doses remains a topic of debate. Some researchers support the existence of a threshold dose below which radiation exposure has no measurable biological effect or could even exert protective effects.^28^ As such, continued surveillance of treated cats will be critical in further evaluating the long-term safety profile of this modality in veterinary patients with careful client counseling, particularly given the relatively young age of most affected cats.

Further research is warranted to validate these findings, including studies with larger sample sizes, inclusion of female cats, incorporation of appropriate control groups, and investigation into optimal dosing strategies and potential protocol adjustments. Generalization of results should be approached with caution until such data are available. Additionally, thoughtful case selection remains essential, as does comprehensive stress management through MEMO. Addressing both the stress-related components and the chronic pain and inflammation associated with cystitis will likely yield the most effective therapeutic outcomes.

In conclusion, this study found that low dose RT appears to be a safe and effective treatment for FIC in male cats with >90% of cats having measurable improvement in clinical signs. Given the central role of stress and chronic pain in the pathophysiology of FIC, treatment approaches that minimize daily stress and systemic drug exposure, such as low-dose RT, could offer a more suitable and sustainable alternative in select cats.

## Supplementary Material

aalaf029_Supplemental_Table_1

## Off-label antimicrobial declaration

The authors declare no off-label use of antimicrobials.

## Institutional animal care and use committee or other approval declaration

Approved by the Clinical Research Review Committee and the Institutional Animal Care and Use Committee (IACUC) of North Caroline State University. Animal Use Protocol IACUC 20-125.

## Abbreviations

CBC: complete blood count
CTRS: central threat response system
FIC: feline idiopathic cystitis
FLUTD: feline lower urinary tract disease
IC/BPS: interstitial cystitis/bladder pain syndrome
LNT: linear no-threshold
LUT: lower urinary tract
MEMO: multimodal environmental modification
NSAID: non-steroidal anti-inflammatory drug
PU: perineal urethrostomy
UA: urinalysis
UO: urethral obstruction
RT: radiation therapy
