# Supplementary material for: Low-dose radiation therapy for idiopathic or interstitial cystitis in male cats
Source: J Vet Intern Med. 2026 Jan 21;40(1):aalaf029. doi: 10.1093/jvimsj/aalaf029 (PMC12881965; doi:10.1093/jvimsj/aalaf029)
Supplement: aalaf029_Supplemental_Table_1 [file aalaf029_supplemental_table_1.docx]

| Cat | Pre-RT UO Episodes | Post-RT UO Episodes | Time to Flare* | Time Free of Signs* | Notes |
| --- | --- | --- | --- | --- | --- |
| 1 | 1 | 0 | 4 | 1303 |  |
| 2 | 4 | 0 | 0 | 1095 |  |
| 3 | 3 | 0 | 0 | 920 |  |
| 4 | 2 | 0 | 0 | 914 |  |
| 5 | 1 | 0 | 395 | 440 |  |
| 6 | 1 | 0 | 152 | 540 | Repeat RT |
| 7 | 2 | 0 | 0 | 616 |  |
| 8 | 2 | 0 | 0 | 580 |  |
| 9 | 3 | 0 | LTF | LTF |  |
| 10 | 1 | 0 | 0 | 497 |  |
| 11 | 2 | 1 | 334 | 150 | Flare with UO |
| 12 | 1 | 0 | 47 | 411 |  |
| 13 | 1 | 0 | 274 | 182 |  |
| 14 | 1 | 0 | 355 | 161 |  |
| 15 | 1 | 0 | 0 | 410 |  |

## Supplementary Table 1. UO episodes before and after RT, time to flare, and time free of clinical signs.

Values are in days unless otherwise specified. LTF = lost to follow-up.
